# Financial health and well-being of rural female caregivers of older adults with chronic illnesses

**DOI:** 10.1186/s12877-025-05853-5

**Published:** 2025-04-10

**Authors:** Nasreen Lalani, Evans Appiah Osei, Siqi Yang, Bhagyashree Katare, Sampada Wagle

**Affiliations:** 1 School of Nursing, Purdue University, West Lafayette, USA; 2 Department of Public Health, Purdue University, West Lafayette, USA; 3 Department of Agricultural Economics, Purdue University, West Lafayette, USA

**Keywords:** Financial stressors, Rural caregiving, Older adults, Self-care, Coping, Wellbeing

## Abstract

**Background:**

Rural caregivers experience significant financial stressors while caring for their older family members with chronic illnesses. Limited access to care, support, and resources in rural areas poses significant financial threats and insecurity for some caregivers. As the majority of rural family caregivers are women, these challenges also represent gender disparities, role imbalances, and division of labor in the society that has rarely been explored in the literature from a rural context. To address these gaps, our study aims to explore the lived experiences of financial burdens and struggles of rural female family caregivers of older adults with chronic illness.

**Method:**

Using a purposive sampling approach, qualitative interviews among *N* = 20 rural woman caregivers of older adults with any serious chronic illness was carried out. Interviews were done in-person, telephone or online as preferred by the participants. Each interview was about 45–60 min. All the data were recorded, transcribed, and analyzed using the thematic content analysis approach.

**Results:**

Our findings showed significant gender role imbalances and financial disparities among the rural women caregivers. Major themes identified were indirect caregiving costs, direct caregiving costs, and barriers in navigating financial support systems. Participants reported losing jobs, experiencing caregiving stress and poor wellbeing, time constraints, financial losses, using pension plan and health coverage benefits to support themselves and their family. Barriers reported include financial decision making and documentation struggles, and difficulties in accessing savings and health coverage benefits and other legal complications.

**Conclusion:**

Rural female caregivers face significant financial threats and insecurities exacerbated by the interplay of gender roles and rural inequities. These inequities need to be addressed to support better caregiving policies and interventions. Provision of financial services and guidance to support rural and disadvantaged women family caregivers in navigating financial resources, financial health planning and decision-making processes is needed. Future comparative and longitudinal studies are recommended to see the long-term effects of financial burdens and inequities on the wellbeing of female caregivers of older adults in the rural communities.

## Background

About 48 million people in the United States (US) are family caregivers who care for their older spouse, parent, or relative at home or other residential care settings [1–3]. Family caregivers also termed as ‘informal’ or ‘unpaid’ caregivers are the ones who are in close supportive relationships with a patient, share illness experiences, participate in most caregiving activities and tasks, and are emotionally and spiritually engaged with their patients. More than 60% of these unpaid or family caregivers are women and provide 75–90% of home-based care to their older family members [2]. For many carers, financial pressures are the most serious concern and can substantially impact their caregiving roles and abilities [1]. The financial costs of caregiving are not limited to out-of-pocket costs but also include caregiver’s time costs and work-related costs such as changes in employment, lost economic opportunities, and reduced productivity at personal, family and professional levels [1, 4]. With the current inflation and loss of jobs, this burden is consistently rising. The financial burdens result in the poor health and wellbeing of caregivers [1, 5–8] and therefore need immediate attention.

The financial burden of caregiving further intensifies among rural and disadvantaged populations due to existing rural health disparities, increased migration, lack of job opportunities and limited access to healthcare services and resources [9]. Women are expected to take the extended role of caregiving to older adults because of the many societal and gender expectations and values. These concerns and challenges are not limited to the US only. Studies found in rural regions of Canada also report similar caregiving role sacrifices and financial strains among the female caregivers of older adults [10, 11]. A study conducted in the Czech Republic also found that a larger percentage of spousal caregivers were women aged 51 or older, and these caregivers tended to spend more time providing care, which was associated with higher caregiving costs and poorer well-being.[12]. Similarly, studies conducted in developing countries such as Ghana, Sudan, and Ethiopia report that informal caregivers of older adults face economic difficulties, higher inflation, a lack of financial services and advice, all of which contribute to emotional distress and poor well-being. [13–16].

The economic value of caregiving and its impact on the well-being of female family caregivers, particularly in rural areas, remains understudied. While a few quantitative studies have measured the costs of caregiving, caregiving intensity, and the time spent by caregivers of older adults [17, 18], the ways in which these caregiving roles contribute to gender and power imbalances—and affect the lives of women, especially in rural and disadvantaged populations—have yet to be addressed. The social and economic impacts of caregiving need a more in-depth understanding from a socioecological and gender equity standpoint to address the financial burdens of caregiving and its effects on the overall selfcare and quality of life of rural female caregivers in a more holistic manner. Given above, our study aims to explore the lived experiences of financial burdens and struggles of rural female family caregivers of older adults.

## Methods

### Study design and setting

The following study is part of a larger explanatory sequential mixed-method study that examined the physical, social, economic, and spiritual impacts of caregiving on the self-care, resilience, and overall well-being of rural female caregivers of older adults with chronic illness [17]. This paper particularly focuses on the qualitative descriptive data gathered about the financial and economic hardships experienced among rural female caregivers caring for their older adults. The study was conducted in the rural counties of Indiana, US. Indiana has a population of 6.87 million, 21.9% of whom reside in rural areas [19]. Indiana has only 33 critical access hospitals and no rural emergency hospitals, highlighting significant healthcare infrastructure challenges in these regions. Rural Indiana is primarily an agricultural area, characterized by farming-based economies that are vulnerable to job loss and economic decline [20, 21]. These areas have a disproportionately higher population of older adults with multiple chronic illnesses, including heart disease, cancer, and dementia. Consequently, there is an increased caregiving burden, lost productivity, and higher healthcare costs [22].

### Recruitment, sample, and data collection

We conducted qualitative interviews among a purposive sample of *n* = 20 rural female caregivers caring for an older family member with any chronic illness. Eligibility criteria include: Women aged 18 years and older who are unpaid family caregivers including as spouse, parent, sibling, neighbor or friend, providing care to an older family member aged 60 or older with any chronic illness or disability for more than six months, at home or any other residential care facility. Participants were recruited in collaboration with community partners, including rural hospitals, clinics, and rural extension partners. Recruitment flyers were posted in the clinics, hospitals, public libraries, churches and rural extension newsletters. In-person recruitment was also done at the rural community fairs and events. Data collection took place from December 2023 to May 2024. To collect the data, an interview guide was developed to elicit caregiving experiences about their financial stressors/challenges and their impacts on female caregivers’ self-care and well-being. (Refer to Table 1). Data was collected through in-person (2), telephone (2), or online interviews (16) using Zoom or Teams online platforms as preferred by the participants. Participants’ demographic data was collected before the interviews. Each interview was about 45-60 min in duration. All the interviews were conducted by the PI along with a trained graduate research assistant. Participants were compensated with a $25 Amazon e-gift card for their time and effort in the study. Ethical approval was obtained from the Institutional Review Board of the university (IRB# 2020–150). Before each interview, study information was provided, and verbal consent was obtained and recorded from the participants. The anonymity and confidentiality of the data were assured. The data was recorded, transcribed, and de-identified by removing all personal identifiers. After de-identification, it was securely stored in an encrypted institutional folder and shared exclusively with the research team.

**Table 1:**
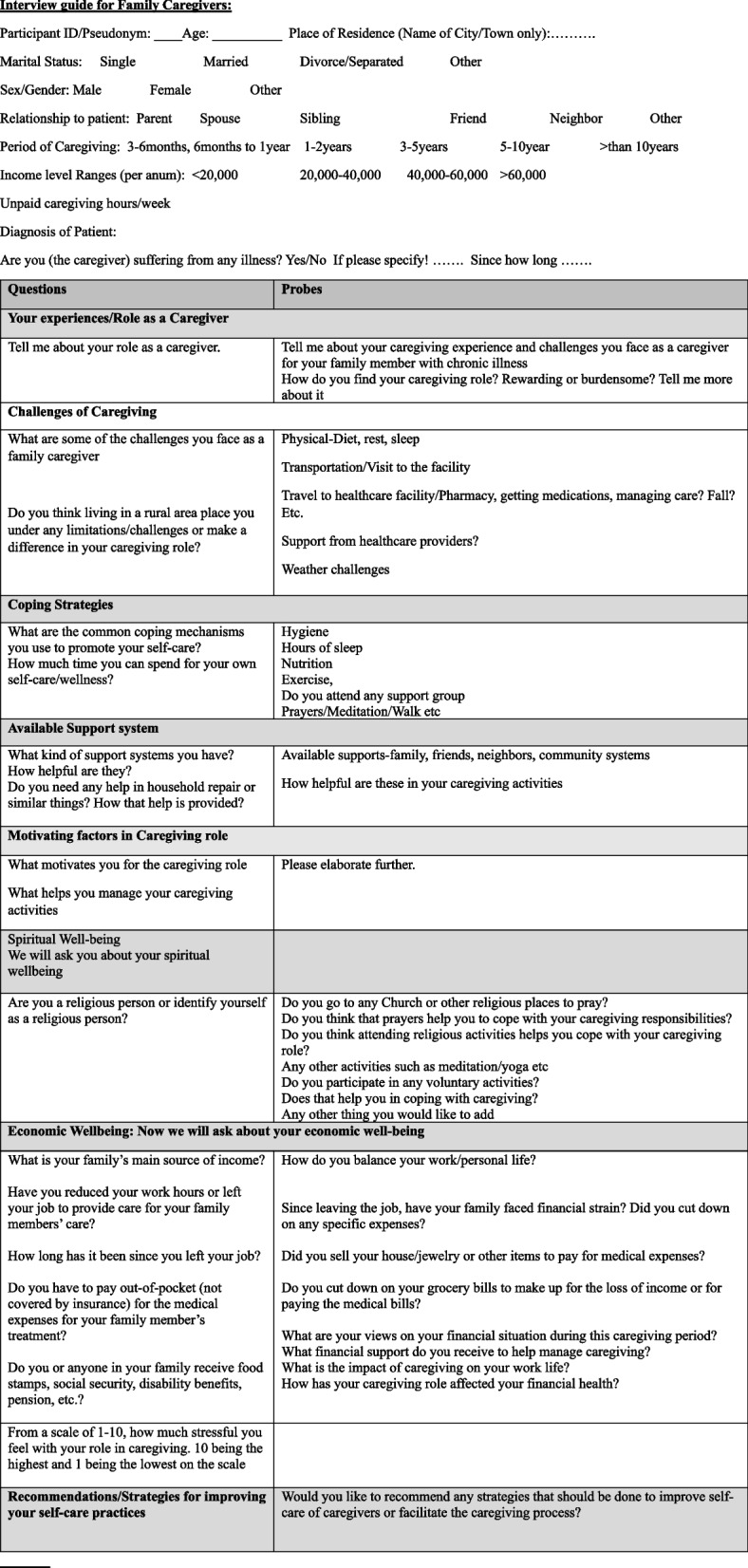
Interview guide for Family Caregivers

### Data analysis

All the data was recorded and transcribed verbatim. Data analysis began upon completion of the initial six interviews for further modification, looking for elaboration on further concepts or categories in the following interviews and assessing data saturation. Data saturation was achieved upon completion of twenty interviews. Data was analyzed using deductive and inductive analysis approaches grounded in the socio-ecological model conceptual framework. This framework highlights how human behavior is shaped by multiple factors, including individual, interpersonal, organizational, community, and policy influences [23]. It is relevant to financial health, as it is impacted by personal finances, family support, community resources, and policies affecting the cost of care. Data was analyzed manually. The analytical process involved several stages: familiarizing with the data, generating initial codes, searching for themes, reviewing these themes, and defining and naming the themes and subthemes [23]. The research team comprised of four research assistants and two faculty members as PI and Co-PI under the study. They read and summarized all the transcripts to gain a comprehensive understanding and interpretation of the quotes, constructs, and conceptual ideas emerging from the data. Upon several iterations, code and categories were formulated and subthemes and themes were assigned. A consensus was obtained by the research team before formalizing the final sub-themes and themes under the data after several research team meetings. Table 2 below illustrates the step-by-step process of theme generation.

**Table 2:** Step-by-Step Coding and Theme Generation (Coding Tree)

Stages of Analysis	Description
Familiarization	All five authors thoroughly reviewed the transcripts multiple times to familiarize themselves with the content and to gain a deeper understanding of the participants' narratives. 4–5 meetings were held to discuss the transcripts among the researchers before proceeding with codes. No follow-up interviews were conducted
Condensation/Condensed Unit	Example of Quotes: “There are times when I'm in the middle of a job or my project, and I have to set that aside to attend to her needs if something urgent comes up. Sometimes I need to be at the hospital for about 8 h each day.” “I took a whole month off from work when he had the transplant” “I can only work part-time. My earnings significantly decreased from $75 an hour to $15 an hour after taxes”
Generating initial codes	Disruption in work, taking out time for hospital stays, absence/leaves from work, work part time, lost hours and income
Categorization/categories	Caregiving-related work/job disruptions, Increased absence and decreased job productivity, lost income
Theme	Indirect Costs of Caregiving

### Rigor

To ensure methodological rigor, the transcription was executed verbatim, and participant recruitment adhered strictly to the inclusion criteria under the study. Participant anonymity was maintained using pseudonyms; each participant was designated as 'R' followed by a sequential number corresponding to their interview order, ranging from R1 to R20. Furthermore, the entire manuscript underwent a thorough review by all authors to resolve any disagreements and reach a consensus. Field notes were taken to capture non-verbal cues from participants, and other environmental cues to help clarify portions of the data and ensure rigor in the findings.

## Results

Participants’ demographic data shows that the majority of the female caregivers were more than 50 years old and above (85%), White (90%) and Black African Americans (10%), married (70%). Most of them were providing care to their parents or in-laws (85%) for more than 6 years (40%) and were devoting more than 20 h/week (45%). More than half (55%) of the caregivers had their income levels less than 60,000/year. (Refer to Table 3.)

**Table 3:** Socio-demographic profile of participants

**Variable**	**Frequency ***N* = 20	**Percentage **100%
**Age Range**		
40–49	3	15
50–59	8	40
60 above	9	45
**Marital Status**		
Married	14	70
Single	4	20
Widowed	2	10
**Caregiver-Patient relationship**		
Daughters/daughter in laws	17	85
Spouse	3	15
**Income Levels**		
Below 20,000	4	20
**20,000–60,000**	6	30
**60,000 and above**	10	50
**Care giving Duration**		
**0–2 years**	8	40
**3–5 years**	4	20
**6 + years**	8	40
**Caregiving hours**		
**0–20 h/week**	11	55
**21 + hours/week**	9	45

## Study Themes:

The thematic analysis of the study yielded three overarching themes, each encompassing relevant subthemes. Three major themes identified were indirect costs of caregiving, direct costs of caregiving, and barriers in navigating financial support systems. The subthemes included lost employment, benefits and career goals, caregiver distress and poor wellbeing, time costs, financial losses, using pension plans and health coverage benefits, financial decision making and documentation struggles, and difficulties in accessing savings and health coverage benefits. (Refer to Table 4).

**Table 4:** Themes, Subthemes and codes

Themes	Subthemes	Codes
Indirect Costs of Caregiving	a. lost employment, benefits, and career goals b. caregiver distress and poor wellbeing c. time costs	work disruption, prolonged hospital stays, leave, absences, part-time employment, career sacrifice, put work on hold stress eater, eat to cope, felt working under crises, sleep disruptions, no relaxation time home bound, no time to self, no personal life, had to be present at home always
Direct Costs of Caregiving	a. financial Consequences b. using pension plans and health coverage benefits	difficulty paying bills, using retirement money and savings, cutting down expenses on meals and groceries, guilt of not being able to fulfil wishes of dying care recipient pension pay, rely on income, rely on retirement savings, social security benefits
Barriers in navigating financial support systems	a. financial decision making and documentation struggles b. difficulties in accessing savings and health coverage benefits	no help or support in making decisions, lack of guidance on Medicare and Medicaid, insurance, and healthcare benefits, complex paperwork difficult to access funds, completing transactions alone, legal complications, technical issues, uncertainty about future

## Theme 1: Indirect cost of caregiving

Participants reported several indirect costs including lost employment and work productivity, moral distress, and time costs that directly or indirectly added to their social and economic suffering resulting in poor selfcare, coping, and wellbeing.

## Sub-theme 1: Lost employment, benefits, and career goals

Most caregivers reported that due to overwhelming caregiving roles and responsibilities, they had to either reduce their working hours, step down from full-time to part-time, leave jobs, sacrifice their career goals, or be forced to take early retirements. While giving care to the ill older family members at home, they had to look after their job responsibilities, housework and childcare. Due to multiple tasks at hand, they were forced to take leave of absence from work or put aside their job responsibilities causing lost productivity and household income. One of the participants, a retired university professor taking care of her father with dementia said:

“I currently work part-time for an auction company, handling their online listings for auctions. There are times when I'm in the middle of a job or my project, and I have to set that aside to attend to his needs if something urgent comes up. Sometimes I need to be at the hospital for about 8 hours each day…. I had no choice but to put my work responsibilities on hold for the entire month” (R 4, 66 years)

Another participant reported similar experience as:

“I took a whole month off from work when he had the transplant. I was still working through… caring at hospital and home, pay the bills and all other stuff” (R 8, 64 years).

Participants reported that they had limited choices, either they had to reduce their working hours or accept low-paying jobs which in turn resulted in financial strain on the family. As one of them stated:

“Due to my caregiving responsibilities, I can only work part-time. My earnings significantly decreased from $75 an hour to $15 an hour after taxes. How am I supposed to cover these bills? " (R 1, 44 years)

“In this rural area, job opportunities are scarce, so we take whatever work is available, including farming, to support ourselves financially for now.” (R 18, 56 years)

There were few who had to sacrifice their career goals and aspirations to fulfill the caregiving roles or reduce their working hours. A participant, who had a fulfilling career as a dentist earning a decent amount of income for the family said that she had to sacrifice her dream career job and take another low-paying job to provide care for her chronically ill mother at home. Due to several financial hardships, caregivers had to cut down on their meals and other household expenses. Below are quotes from the participants:

“I had to give up my job and my career to care for my parent and become a homemaker instead.” (R 9, 50 years)

“Due to the caregiving, I have reduced the hours for my small business….I had to cut down on food and daily expenses to be able to support him financially” (R 18, 56 years)

However, one participant remained committed to her career goals but had to relocate closer to the care recipient, renting an apartment near the nursing home. This move significantly increased her expenses due to relocation costs and extended travel time to work.

"I rented an apartment close to the healthcare facility, which also required an hour of travel for work which resulted to extra expenses” (R 8, 64 years)

Some participants were forced to take early retirements and lost their benefits due to increasing caregiving demands.

"I worked as a part-time yoga instructor. Due to increasing caregiving demands, I was not able to continue with my job responsibilities and had to retire earlier than expected." (R 6, 73 years)

## Sub-theme 2: Caregiver distress and poor wellbeing

Female caregivers reported going through several financial hardships and experiencing increased amount of stress juggling with multiple priorities and caregiving roles. Due to the financial hardships, participants reported not having adequate sleep and diet, inability to cope with their selfcare and wellbeing. Participants reported feeling like they are constantly ‘*functioning under crisis’* and it is overwhelming for the whole family. One of the female caregivers providing care for her mother with multiple chronic illnesses reported as:

“Her condition is getting worse. She has congestive heart failure and now her dementia is also beginning to progress. This is getting overwhelming. The three other family members and I take turns doing whatever needs to be done. It often feels like we’re constantly operating in crisis mode, always managing one urgent situation after another. It's like functioning underneath crisis. We feel that we are constantly functioning in a crisis mode.” (R 5, 54 years)

As described earlier, increasing caregiving demands and expectations were also causing significant disruptions in caregivers' dietary habits and sleep patterns. Almost all participants reported poor eating habits, no time to prepare regular meals, and sleep deprivation.

“I am a stress eater…. I just eat to cope. Rarely, I find time to prepare my own meals and rely on whatever is available.” (R16, 64 years)

Regarding sleep deprivation, one of the participants caring for her spouse with dementia said:

“some days he would get up at 1:00 am, go to the living room and turn the TV on very loud because like I said, he couldn't hear…. I would have to shut the bedroom door so that I could go back to sleep. But even then, you're not asleep, because at any moment he's going to call your name or ask for something.” (R2, 72 years)

Another participant taking care of her older mother with dementia said that:

“My mind is always racing. I can’t sleep because am always worried and thinking about her. And if I'm not worried about her, I'm worried about other things such as work and other stuff.” (R 5, 45 years).

## Sub-theme 3: Time costs

Time was identified as a major indirect cost of care affecting female family caregivers’ wellbeing and quality of life. Participants reported an inability to find time for themselves as well as their families as a whole. For some, this time was overwhelming and stressful whereas for few, it was perceived as part of their role commitment and meaningful. One of the participants caring for her spouse with cancer and dementia reported being homebound for the last three years until her spouse passed away recently. She reported that this was her personal choice out of her marital commitment and devotion towards her spouse and found it meaningful. She said that:

I couldn't do much because the worse he got, the more he didn't want me to leave the house, even to get to the mail. He was so insecure without me. Even if I was in the kitchen and he was in the living room, he would keep asking, where are you? What are you doing? If I go for a bath, he will call me…. I knew he wasn't going to last forever. And I thought I'll just stay home until he's gone.” (R2, 72 years).

Another participant, a single parent with two children and a social worker by profession, caring for both of her parents with serious chronic illness and disability reported similar struggles.

" With all these multiple caring roles, I do not have any time left to myself. I don't have any personal life. It's like what is a personal life. I just do what I need to do.” (R5, 45 years).

## Theme 2: Direct costs of caregiving

The direct financial costs associated with caregiving included financial losses, insufficient funds to cover healthcare costs, out of pocket expenses, hospital bills, debts, and losing personal and family savings and benefits if any. The following are the sub-themes:

## Sub-theme 1: Financial Consequences

Consider this: Some participants reported financial losses due to long-term caregiving, struggling to make ends meet and pay bills. Barriers such as the inability to take a full-time job, reduced work hours, working from home, limited access to healthcare, insufficient caregiving benefits, and early retirement contributed to mounting debts and financial strain. The following quotes illustrate these experiences:

"How am I supposed to pay for this bill? What am I supposed to do? Lately, we had to cash out our retirement savings early…. I just don't know what else to do." (R 14, 63 years)

"In our culture, we tend to put everything into our 401(k) retirement savings, which isn’t necessarily a bad thing... but what if you can’t rely on it? Finances have been really tough, especially since my husband earns only about $45,000 a year." (R1, 44 years).

Some caregivers felt bad about either overspending or not being able to spend enough to fulfil their care recipients wishes while providing care at the end of life. Below are the supporting quotes:

“We spent some money on the credit card eating out more frequently than we should have.” (R 2, 72 years)

“He wanted to do things, and if I said we don't have the money, he becomes agitated.” (R 2, 72 years)

## Sub-theme 2: Using Pension Plans and Health Coverage Benefits to Offset Caregiving Costs

While many caregivers experienced significant financial strain, a few participants were able to alleviate the direct costs of caregiving by relying on their parents' or spouse’s retirement funds, pension pay, Medicare benefits, insurance policies, and Social Security. These financial resources helped them manage healthcare expenses and reduce out-of-pocket costs, ultimately easing their caregiving burden. “The finances have stayed the same, my parents are pretty okay with their retirement. So, we don't live wild lives. But we're okay. We have enough finances.” (R 13, 58 years)

"He generally has sufficient funds to cover his medical expenses and manage his retirement needs. We are glad to provide financial assistance, when necessary, though this occurs infrequently—perhaps two or three times." (R 14, 63 years)

“I did not face financial strain or cut down any daily expenses. My husband was on disability benefits prior to his transplant. And then after two years, you automatically go on to Social Security. So, he gets Social Security benefits and then he has many investments that he's made.” (R 8, 64 years)

## Theme 3: Barriers to navigating financial support systems

Navigating healthcare and financial systems was challenging for the female caregivers of older adults in the rural areas. Such challenges include but are not limited to securing adequate financial support and resources, and proper documentation. These are discussed below under the following sub-themes.

## Subtheme 1: Financial decision making and documentation struggles

Participants found it overwhelming navigating financial systems and resources and making appropriate financial decisions during the caregiving process. They found difficulties understanding various terminologies, completing and signing healthcare coverage and benefits and related financial documents for their care recipients. Participants reported that they needed appropriate support and advice in making the right financial choices and decisions on several occasions while providing care for their loved ones. Below are some quotes:

"Navigating Medicare was challenging—I struggled to understand what it covers, what it doesn’t, and how secondary health insurance fits in. Even the terminology used was difficult to figure out." (R15, 59 years)

When it comes to making financial decisions or discussing things like the house or future plans, I have to handle everything on my own, which is quite stressful. Being responsible for everything has been overwhelming. (R 6, 73 years)

“Do I need to switch to Medicaid or Medicare? What should I do about the taxes that weren't filed….it was hard to figure out who to talk to”. (R 9, 50 years)

### Subtheme 2: Difficulties in accessing savings and health coverage benefits

Some female caregivers reported enormous struggles to access their healthcare saving funds, insurance policies or benefits for their dying spouse or older parents due to frozen saving accounts, and restrictive legal policies and procedures. One of the participants caring for her spouse with late stages of brain cancer reported that:

“Although the insurance policy premium was due and I had sufficient funds, I couldn’t complete necessary transaction…. And I said, well, I want to pay for this long-term care we've been paying for all these years, but they won't let me talk to them without the power of attorney. …. My spouse was unconscious and not able to make decisions or sign…. They wanted a note from doctor and it was a huge struggle then….” (R 7, 74 years)

Another participant described the challenges she faced in obtaining health insurance for her spouse and expressed skepticism about the financial assistance advertisements and other marketing strategies to support older people. She added:

"He did not have life insurance. He had been a heart patient for over 20 years, so I was really not able to get life insurance for him. I know those advertisements on TV sound good, but they only offer about $2,000." (R 2, 72 years)

Rural female family caregivers needed better services and guidance to cope with their financial struggles and challenges and provide quality care for their older adults and families.

## Discussion

Our study presents significant findings on the economic value of caregiving. It discusses several financial burdens/hardships, barriers to navigating financial resources and decision-making, and ultimately, their impact on the financial wellbeing of rural female family caregivers of older adults with chronic illnesses or disabilities. Rather than viewing financial hardships and wellbeing in monetary terms only, our study adopts a broader gender and health equity perspective to underline the economic value of caregiving. It highlights practical and policy implications for overcoming financial disparities, improving access, and enhancing the financial well-being and quality of life of disadvantaged female family caregivers of older adults in rural areas.

Our demographic findings indicate that most participants were married and providing care for their older parents or in-laws. Over half of the caregivers were aged 50 years or older and spent more than 20 h/week providing care. Previous studies have also demonstrated that gender roles and family values are critical in caregiving [24, 25]. Similarly, our findings suggest that many caregivers took on this role due to cultural expectations, familial duty, and limited alternative care options. It is often the women who take the major responsibility of caregiving and tend to sacrifice their careers, jobs and aspirations to care for their older family members, including parents, siblings or spouses with any illness or disability [26]. The tendency for women to take on the primary caregiving role often stems from deeply ingrained societal and cultural norms that position women as the nurturers within the family, with caregiving seen as an extension of their gendered responsibilities [27, 28]. Additionally, economic factors—such as the prevalence of women in lower-paying or more flexible jobs—further exacerbate this dynamic, leaving many women with limited choices but to prioritize caregiving roles at the expense of their careers and personal aspirations. As a result, nearly 21% of women experience financial strain, and 40% reduce or cease saving for their future [29–31].

The financial risks and insecurities, lost income, reduced career opportunities, early retirements and diminished savings make rural female caregivers more vulnerable to significant physical and psychological stress and harmful behaviors compared to their male counterparts [32–35]. The study participants also suffered a lack of personal and social life, inadequate diet and sleep, and no time for self-care, resulting in poor family functioning and reduced quality of life. These findings are consistent with other studies [36, 37]. Consistent with previous findings, rurality can be a major factor contributing to the poor financial and emotional well-being of female family caregivers given the lack of support systems and resources [38, 39]. While policies such as the Family and Medical Leave Act (FMLA) and paid sick leave has shown to assist family caregivers in managing financial stress [40] disparities in access to these benefits exist particularly among working women, low-wage workers, and less-educated employees. These groups are less likely to benefit from paid sick leave and family leave further exacerbating their challenges.

The study findings also highlight several barriers among caregivers in handling complex billing systems, accessing their financial accounts, completing documentation, and other legal complications. A lack of support and guidance in financial planning and decision-making was also reported. These barriers are commonly reported in rural and remote areas, given the scarcity of human and material resources [41–44]. The complex nature of the existing healthcare coverage policies and benefits often makes it challenging for caregivers to navigate appropriate resources in planning and making adequate financial choices. They spend long hours dealing with insurance companies, filling out forms, and resolving billing disputes with limited support. Errors in coding and billing can lead to claim denials, underpayments, and compliance issues, which can be overwhelming and time-consuming for the caregivers [45–47]. Immediate attention is needed to promote the financial literacy and support financial healthcare planning and decision making among rural female caregivers caring for their older adults with long-term serious illnesses.

### Strengths and limitations

Our study informs several financial and gender disparities from a rural caregiving context and advocates for better financial support and caregiving policies to improve the holistic wellbeing and quality of life of rural female family caregivers of older adults. It is noteworthy that the study results are only limited to the women and most of them were White. There is a likelihood of social desirability in the participants’ responses in the interviews. Future studies can be extended to compare caregivers of different genders, race and ethnicity, geographic regions, or types such as formal/paid or informal/unpaid caregivers to broaden the scope and gather a comprehensive understanding of the studied phenomena.

## Conclusion

Rural female caregivers face significant financial threats and insecurities while caring for their older family members, a situation that is often exacerbated by the interplay of gender roles and rural inequities. Gender expectations, which often place caregiving responsibilities predominantly on women, can lead to a disproportionate financial burden, particularly in rural settings where access to resources and support systems is limited. These inequities and role imbalances need to be addressed in a rural caregiving context to support better caregiving policies and interventions. Better financial services and policies should be in place to support rural and disadvantaged female family caregivers in navigating financial resources, financial health planning and decision-making. This will improve the holistic wellbeing of female caregivers of older adults and ensure an equitable and thriving rural community.

## Recommendations

Caregiver respite support programs should be made available in the rural areas. Polices need to consider the existing gender disparities and role imbalance and its spillover effects on health and wellbeing of rural female caregivers of older adults. Better systems and resources should be made available to assist rural caregivers in billing processes, completing financial documents and/or any legal documentation during hospitalization if needed. Employment leaves and benefit polices need to consider the caregiving challenges of working women, low-wage workers, and less-educated employees and should be accessible to this group. Funding support for the programs is needed to promote financial literacy, financial choices, and decision making in the rural communities. Future longitudinal studies are recommended to examine the long-term impact of financial and gender inequities the caregiving roles and well-being of rural female caregivers of older adults.

## Data Availability

The supporting data can be found within the study.

## Abbreviations

FMLA: Family and Medical Leave Act
IRB: Institutional Review Board
US: United States
